# Melatonin Receptors: A Key Mediator in Animal Reproduction

**DOI:** 10.3390/vetsci9070309

**Published:** 2022-06-22

**Authors:** Yuan Gao, Shuqin Zhao, Yong Zhang, Quanwei Zhang

**Affiliations:** 1 College of Life Science and Technology, Gansu Agricultural University, Lanzhou 730070, China; 2 Gansu Key Laboratory of Animal Generational Physiology and Reproductive Regulation, Lanzhou 730070, China; zhaosq@gsau.edu.cn (S.Z.); zhychy@163.com (Y.Z.)

**Keywords:** melatonin receptors, animal reproduction, rhythm

## Abstract

**Simple Summary:**

Animal reproduction is closely related to economic benefits and food safety in domestic animals, as well as human fertility. There are many factors that affect the reproductive ability of animals, among which studies have confirmed that melatonin can affect animal reproduction. Melatonin receptors play important roles in mediating melatonin signaling, and they are expected to become a drug target for the improvement of animal reproduction capacity and treatment of reproductive diseases. In this review, we briefly summarize melatonin receptors MT1- and MT2-mediated signal transduction, as well as their role in reproductive regulation, including their effects on gametogenesis, gamete quality, reproductive rhythm, endocrine function, and embryonic development. We also briefly summarize the developments in pharmacological research regarding melatonin receptors as drug targets, which can provide a future perspective for the development of drugs and therapeutic methods based on melatonin receptors or their mediated signaling pathways.

**Abstract:**

Melatonin, a hormone produced by the mammalian pineal gland, influences various physiological activities, many of which are related to animal reproduction, including neuroendocrine function, rhythm regulation, seasonal behavior, gonadogenesis, gamete development and maturation, sexual maturation, and thermoregulation. Melatonin exerts beneficial actions mainly via binding with G-protein-coupled receptors (GPCR), termed MT1 and MT2. Melatonin receptors are crucial for mediating animal reproduction. This paper reviews the characteristics of melatonin receptors including MT1 and MT2, as well as their roles in mediating signal transduction and biological effects, with a focus on their function in animal reproduction. In addition, we briefly summarize the developments in pharmacological research regarding melatonin receptors as drug targets. It is expected that this review will provide a reference for further exploration and unveiling of melatonin receptor function in reproductive regulation.

## 1. Introduction

Melatonin (*N*-acetyl-5-methoxytryptamine) is a quite ancient molecule in evolutionary history, which is produced in many eukaryotes and prokaryotes. In vertebrates, the pineal gland produces melatonin in a circadian pattern and in synchronization with the dark phase of the environmental light/dark cycle. The secretion of melatonin affects various physiological activities, such as antioxidation, thermoregulation, immunoregulation, neuroendocrine function, sexual maturation, regulation of seasonal reproduction, and certain aspects of aging [1,2,3].

**Figure 1:**
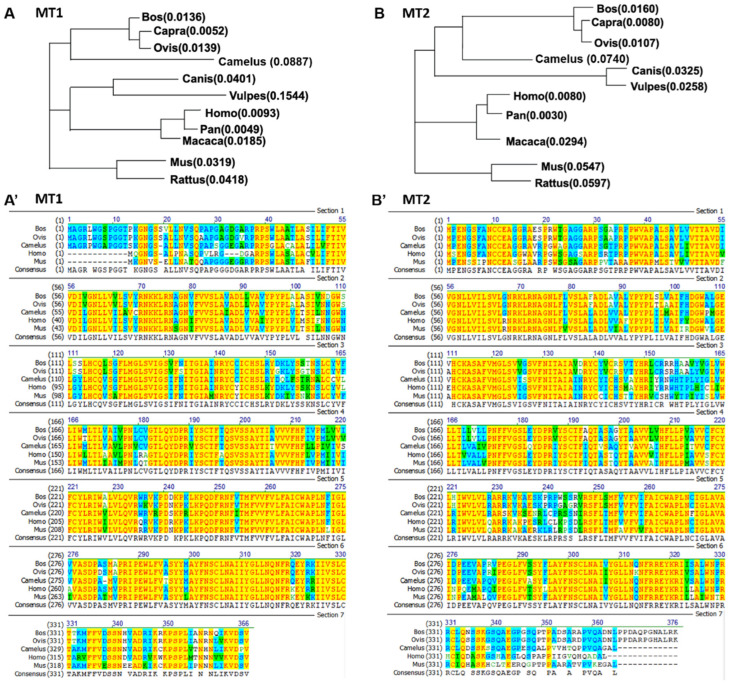
Homology alignment and phylogenetic tree of MT1 and MT2 in mammals. (**A**) Phylogenetic tree of MT1 in mammals. (**A’**) Homology alignment of MT1 in mammals including bovine (Bos), sheep (Ovis), camel (Camelus), human (Homo), and mouse (Mus). (**B**) Phylogenetic tree of MT2 in mammals. (**B’**) Homology alignment of MT2 in mammals including bovine (Bos), sheep (Ovis), camel (Camelus), human (Homo), and mouse (Mus).

Melatonin exerts its biological effects by combining with specific and high-affinity melatonin receptors. In animals, there are two melatonin receptors: MT1 (also called Mel1a, gene officially named MTNR1A) and MT2 (also called Mel1b, gene officially named MTNR1B). The melatonin receptor family belongs to the class A rhodopsin type G-protein-coupled receptor (GPCR) subfamily [4,5]. The human MT1 receptor is composed of 350 amino acids, while the MT2 receptor is composed of 362 amino acids, and they show 55% and 70% homology in the transmembrane domain [6]. Interestingly, ruminants such as cow, sheep, and camel have MT1 and MT2 receptors that are about 10 amino acids longer than those in human [7] (Figure 1), although there is no report of significant functional differences among species. The two receptors have 60% homology among species, including humans, rats, mice, and ruminants [1,8,9]. An additional member, MT3, also termed Mel1c, has been identified but only in nonmammalian species, such as birds, amphibians, and fish [10,11].

A related protein, GPR50, found in eutherian mammals, is also classified as a member of the melatonin receptor family due to its high sequence homology (50%) to MT1 and MT2, which is the ortholog of Mel1c in mammals [11]. However, GPR50 is usually considered as an orphan GPCR because no reports have shown its direct binding to any known ligand, including melatonin [12]. The homolog GPR50 is, thus, instead regarded as a melatonin receptor-related regulatory protein. This review focuses on the function of melatonin receptors MT1 and MT2, particularly their roles in animal reproduction.

## 2. Melatonin Receptor-Mediated Signal Transduction

**Figure 2:**
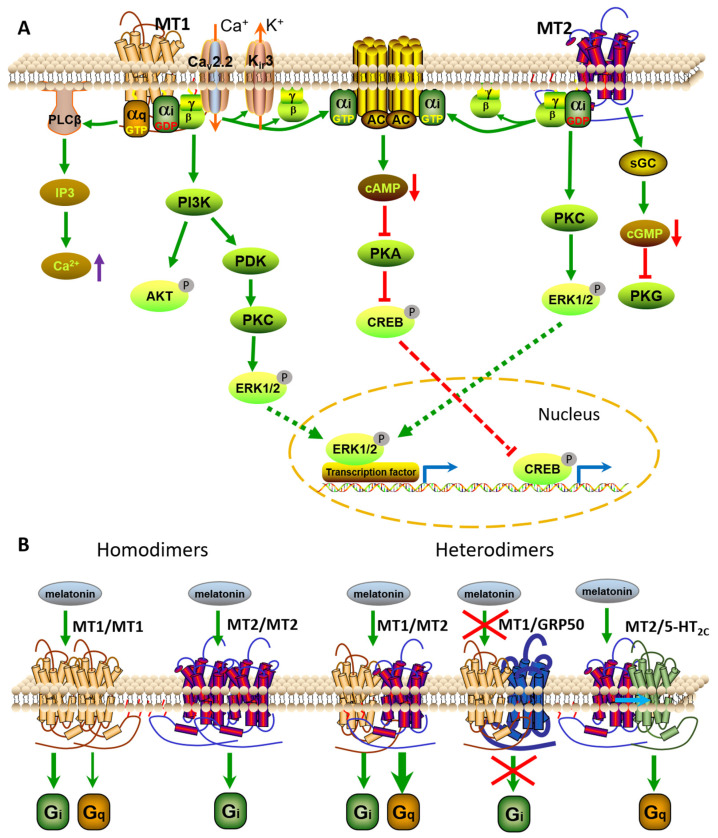
Melatonin receptor signaling pathways. (**A**) Both melatonin receptors MT1 and MT2 trigger toxin-sensitive Gαi and -insensitive Gq proteins, decreasing cAMP levels, protein kinase A (PKA) signaling, and CREB phosphorylation. The MT1 receptor also stimulates Gβγ-dependent PI3K/Akt and PKC/ERK pathways, in addition to activating the Gq-dependent PLC pathway and increasing intracellular levels of Ca^2+^. Moreover, MT1 mediates the activation of K^+^ channels (Kir3) and Ca^2+^ channels (Cav2.2). The MT2 receptor also stimulates Gαi-dependent PKC/ERK signaling and decreases intracellular cGMP levels. (**B**) Melatonin receptor homodimer and heterodimer signaling. MT1/MT1 homodimers mediate intracellular signaling mainly via the Gi pathway rather than the Gq pathway, while MT2/MT2 homodimers signal exclusively via the Gi-dependent pathway. MT1/MT2 heterodimers mainly trigger Gq activation rather than Gi signal. The formation of the MT1/GPR50 dimer prevents the binding of MT1 to melatonin and blocks Gi signaling. The MT2/5-HT_2c_ heterodimer stimulates 5-HT_2C_-mediated Gq signaling through MT2 transactivation. Kir3, G-protein-coupled inwardly rectifying potassium channel; Cav2.2, voltage-gated calcium channel; CREB, cAMP-responsive element binding; sGC, soluble GC.

Melatonin receptors including MT1 and MT2 are classic GPCRs that stimulate downstream signaling by activating G proteins. Although the intrinsic affinity of MT1 and MT2 receptors for different G proteins is still not fully determined, current research shows that both MT1 and MT2 receptors mainly interact with G_i_ proteins, including Gα_i2_ and Gα_i3_ proteins, and G_q/11_, but not with Gα_i1_, Gα_z_, Gα_o_, Gα_12_, or Gα_s_, thus resulting in inhibition of adenylate cyclase (AC) activity, forskolin-stimulated cAMP, and protein kinase A signaling. Other reports have revealed that both MT1 and MT2 receptors bind to Gα_16_ to potentiate melatonin signaling through the JNK pathway in COS-7 cells [13]. In addition to modification of cAMP levels, melatonin receptors can modulate levels of diacylglycerol, inositol trisphosphate, and Ca^2+^ in a cell context-dependent manner [14] (Figure 2A).

Melatonin can also regulate ion channels via signaling pathways mediated by its receptors. Melatonin regulates muscle contractile responses in arteries by modifying the activity of large-conductance calcium-activated potassium channels (BK_Ca_ or termed K_Ca_1.1) [15]. Further studies have indicated that melatonin-induced modulation of BK_Ca_ channels occurs mainly through activation of both G_i_/cAMP/PKA and G_q_/PLC/Ca^2+^ signaling pathways in the myometrium (Figure 2A).

In terms of transcriptional regulation, melatonin signaling typically inhibits cAMP-responsive element binding (CREB), which activates gene transcription though the ERK pathway. It is currently understood that MT1 and MT2 receptors differ primarily at the signaling level in that they inhibit cGMP production [16] (Figure 2A).

### 2.1. Melatonin Receptor Oligomers

Similarly to other GPCRs, MT1 and MT2 receptors can also exist as homodimers and/or heterodimers with themselves or other GPCRs in cells [17,18] (Figure 2B). The formation of MT1/MT1 homodimers occurs 3–4-fold more often than that of MT2/MT2 homodimers and MT1/MT2 heterodimers, although no apparent effect of melatonin activation has been observed on the oligomerization state of the receptors. Unlike receptor monomers, these dimers are functionally related. In mouse, MT1/MT2 heterodimers in rod photoreceptor cells play a crucial role in improving retinal light sensitivity via the PKC pathway at night, which is not normally triggered by activation of monomeric MT1 or MT2 receptors [19]. In addition, knockdown of MT1 or MT2 receptors could alter melatonin-directed ERK and Akt pathways at a low concentration, while melatonin decreases forskolin-induced cAMP production only under the condition that the cells express both MT1 and MT2 receptors in cerebellar granular cells [20] (Figure 2B).

The orphan receptor GPR50, a member of the melatonin receptor family, can also heterodimerize constitutively and specifically with MT1 and MT2 receptors [21]. MT1/GPR50 heterodimer formation markedly inhibits MT1 receptor-coupled ligand binding and signal transduction, including melatonin binding, as well as G_i_ protein and β-arrestin coupling of MT1 receptors. On the other hand, the formation of MT2/GPR50 heterodimer does not modify MT2 function [21] (Figure 2B). The physiological function of the MT1/GPR50 heterodimer and the modulators regulating their association and dissociation in vivo are yet to be explored.

MT1 and MT2 receptors are also able to form heterodimers with the serotonin receptor, 5-HT_2C_ [22]. The formation of MT2/5-HT_2C_ heterodimers is more efficient than the formation of 5-HT_2C_ homodimers and MT1/5-HT_2C_ heterodimers. MT2/5-HT_2C_ heterodimers exhibit a 5-HT-mediated G_q_/PLC response. Thus, melatonin activates both G_i_-dependent signaling and G_q_/PLC-mediated signaling in the presence of MT2/5-HT_2C_ heterodimers, which is not observed in only MT2 expressed cells [22]. These reports indicate that the melatonin-activated MT2 receptors are capable of allosteric transactivation of 5-HT_2c_-dependent G_q_ signaling (Figure 2B). These heterodimers were also targeted by the compound agomelatine, an antidepressant drug, suggesting that MT2/5-HT_2c_ heterodimers may be involved in the drug-mediated antidepressant effects.

### 2.2. Melatonin Receptor-Mediated Biological Effects

Melatonin receptors play a key role in various melatonin-mediated physiological activities, including regulation of circadian rhythm, stem-cell survival, maturation, and differentiation, antioxidation, apoptosis, and cancer [23].

Many studies have revealed that both MT1 and MT2 receptors are involved in melatonin-modulated neuronal firing rate and clock gene expression in hypothalamic suprachiasmatic nucleus neurons (SCN) in a G_i_-dependent, but cAMP-independent manner, which constitutes the master clock in mammals [24,25]. The MT1 receptor-mediated influence on firing rate mainly exists through activation of G-protein-coupled inwardly rectifying potassium channels, such as Kir3, while MT2-induced neuronal activity mainly occurs via stimulation of the PKC signaling pathway [26,27]. A report showed that the MT1 receptor regulates neuronal firing rate by inhibiting P-type Ca^2+^ channels in cerebellar Purkinje cells through the G_i_/G_βγ_/PI3K/PKCδ signaling pathway [28]. Melatonin regulates clock gene expression through MT1 receptors in a G_i_-dependent manner in the striatum [29].

MT receptors are also reportedly involved in melatonin-transduced photic information by regulating the expression of clock genes including Clock, Per1, Cry1, and Bmal1 [30,31,32,33]. Current research demonstrates that both receptors are involved in the clock machinery responsive to melatonin in the retina. Knockout of the MT1 receptor significantly affects the rhythmic expression of clock genes Per1 and clock-controlled genes, such as cfos [34,35,36]. Melatonin controls retinal light sensitivity at night as a function of MT1/MT2 heterodimers via activation of the G_q_/PLC/Ca^2+^ pathway and survival-related Akt/FOXO1 signaling pathway [19,37,38]. Although the exact mechanism underlying melatonin’s effects on clock genes has not been determined, the effects of melatonin on rhythmic clock gene expression may be cell type-dependent [39].

Melatonin also modulates stem-cell survival, differentiation, and maturation, and the effect is melatonin receptor-dependent. Reports have shown that melatonin-induced cell regulation can be blocked by the melatonin receptor competitive antagonist, luzindole, in neural stem cells [40]. In addition, the melatonin-induced neural differentiation-related signaling pathway, PI3K/Akt, can be prevented by antagonist luzindole in pluripotent stem cells [41]. However, prolonging the treatment time of embryonic stem cells in melatonin favors the cell’s pluripotency state in an MT1 receptor-dependent manner, due to a synergistic effect between the actions of the PI3K/Akt pathway and ERK pathway [42]. In addition, melatonin protected cerebellar neurons from LPS toxicity in mouse, which could also be blocked by the competitive melatonin receptor antagonist luzindole, and neurons were more prone to cell death in the luzindole-treated and MT1-silenced cells [43,44].

Melatonin receptors are also involved in melatonin-induced antioxidant and antiapoptotic effects [45]. Melatonin modulates the expression of Bax and Bcl-2 proteins, as well as inhibits the release of cytochrome c and the activation of caspase-3. Melatonin-induced Bax/Bcl-2 translocation occurs via the JAK2/STAT3 pathway, and its antiapoptotic effect is mediated by ERK activation and p38 MAPK inhibition [46,47,48]. In addition, melatonin-activated signaling cascades also include activation of sirtuin histone deacetylases (SIRTs) through AMPK/SIRT3/SOD2 and SIRT1/PPAR-γ coactivator (PGC-1α) [49,50]. The regulation of transcription factor PGC-1α is MT1 receptor-dependent [36]. Blockage of melatonin receptors significantly promotes hCG-induced apoptosis in mouse [7].

Melatonin receptor signaling also plays a role in cancer. In general, melatonin has shown antitumoral properties in tumor models, including inhibiting proliferation and inducing apoptosis. Reports have indicated that melatonin inhibits Akt, p38 MAPK, and mTOR signaling in ovarian cancer [51]. In breast cancer, melatonin inhibits tumor growth mainly through MT1 receptor-mediated inhibition of the phosphorylation of signaling molecules such as Akt, PKC, and ERK [52,53], which results in activation of p53, a DNA-protective signal, in a melatonin receptor-dependent manner [54,55].

## 3. Function of Melatonin Receptors in Animal Reproduction

Studies have found that melatonin receptors are expressed in several central nervous and numerous peripheral tissues, including the testis and ovary [4]. In particular, melatonin is involved in modulation of the hypothalamic–pituitary–gonadal (HPG) axis, which is a quite important regulatory center for animal reproduction, both in seasonal breeding animals and in non-seasonally bred animals, including humans [11]. Some investigations have revealed that the MT1 receptor is widely distributed in endocrine tissues and brain regions, which are the main response sites of melatonin-induced physiological and circadian effects. However, the MT2 receptor is generally absent in the mammalian hypothalamus and pituitary gland and is completely not expressed in several seasonally bred rodents [56]. These data indicate that MT1 is the more important receptor for melatonin-modulated reproductive regulation in mammals.

### 3.1. Effects of Melatonin Receptors on Gametogenesis

Male reproductive functions are mainly regulated by the luteinizing hormone (LH) and the follicle-stimulating hormone (FSH) secreted by the pituitary gland. LH functions through binding to Leydig cells in the testicular interstitium and stimulating androgen synthesis, such as testosterone, which is essential for maintaining spermatogenesis and male fertility [57]. FSH targets Sertoli cells in the testis, stimulates their secretion, and affects subsequent spermatogenesis [58]. Both MT1 and MT2 receptors are expressed in testicular cells. Studies have shown that, by binding MT1 receptors, melatonin exerts a direct inhibitory effect on the hCG-induced cAMP signal and testosterone synthesis in Leydig cells via reducing the expression of key steroidogenic genes, including p450scc, p450c17, and StAR [57,59], which are essential for testosterone synthesis [60,61]. Melatonin acting through the MT1 receptor located in Leydig cells stimulates corticotropin-releasing hormone (CRH) production [59], which is a negative modulator of hCG-stimulated testicular steroidogenesis. On the other hand, knockdown of melatonin receptors, especially MT1, can block hCG-stimulated testosterone secretion via inhibiting the expression of steroidogenic genes [7]. On the other hand, melatonin exerts a protective action by mediating steroidogenic enzyme expression and regulating sex steroid production in cadmium (Cd)-induced testicular toxicity. Cd is a heavy metal that induces high testicular toxicity with widespread prevalence in the general population by increasing oxidative stress and apoptosis [62]. These seemingly contradictory results show the multiplicity of melatonin’s functions. Melatonin, as an antioxidant molecule, can protect cells against oxidative damage induced by Cd and other factors, while it also acts via MT1 and MT2-mediated signaling and influences gene expression and hormone secretion.

Reports have shown that Sertoli cells directly regulate testosterone secretion by binding to the MT1 receptor, and melatonin increases the Sertoli cell response to FSH during testicular development [59]. Melatonin acts via its receptors to stimulate the expression of spermatogenesis-related genes, including Pdgfa, Occludin, Dhh, Cyclin D1, Cyclin E, and Claudin [10]. Melatonin also facilitates the expression of glial cell line-derived neurotrophic factor (GDNF) in a receptor-dependent manner to promote proliferation and self-renewal of spermatogonial stem cells (SSCs), which is the basis of spermatogenesis [63]. Moreover, the MT1 and MT2 receptors are involved in melatonin-induced energy metabolism, including the increase in glucose consumption and lactate metabolism [64]. Considering that male fertility and the process of spermatogenesis are strongly dependent on Leydig cells and Sertoli cell function, melatonin receptors have been strongly demonstrated to play an essential role during spermatogenesis regulation (Table 1).

In female animals, the MT1 receptor is widely distributed in the ovary and is crucial in its melatonin-regulated activities, delaying the decline in fertility in female animals [65]. The growth and development of follicles are complicated, involving five stages: primordial follicle, primary follicle, preantral follicle, antral follicle, and mature follicle [67]. Oxides such as ROS produced during follicle formation lead to oocyte damage and follicular atresia. Melatonin, as an antioxidant, can eliminate ROS and attenuate oxidative stress, protect oocytes and granulosa cells, and improve the fertilization rate and pregnancy rate [68]. In addition, melatonin increases the total number of oocytes and their quality, whereby more oocytes with normal morphology can generate more blastocysts after in vitro fertilization. Mechanistic studies have revealed that MT1 and MT2 receptors are detectable in oocytes and granulosa cells and are responsive to estrogen levels during follicle development [75]; moreover, knockout of the MT1 receptor leads to a significant reductions in the number of oocytes, litter size, and expression of Silent information regulator 1 (SIRT1), c-myc, and CHOP in mouse ovaries, demonstrating that the beneficial effects of melatonin on oocytes are mediated by the MT1 receptor and AMPK/SIRT1 signaling cascade [65,76]. Melatonin improves oocyte development ability and fertilization capacity via a receptor-mediated demethylation mechanism, including an increase in Tet1 gene expression and decrease in Dnmt1 gene expression [69]. In addition, the MT1 receptor is crucial in melatonin-mediated protection against ovarian damage induced by cisplatin, a chemical drug that inhibits cell mitosis [77] (Table 1).

### 3.2. Melatonin Receptors and Gamete Quality

Gamete quality is closely related to zygote formation and embryo development, which further influences the productivity of economic animals and the health of human offspring. Melatonin, as an effective agent to improve gamete quality, has received extensive attention. In vivo, subcutaneous implantation of melatonin ameliorated semen quality, including improvements in sperm motility, viability, total motile sperm, and rapid motility in mammals such as rams, bucks, cattle, and buffalos [66]. In vitro, melatonin treatment could significantly reduce the rate of sperm deformity, improve sperm stability, protect sperm viability, and improve the fertilization capacity of sperm, including non-sorted and sex-sorted sperm [78,79]. High-quality frozen sperm is essential in shortening the animal breeding cycle and factory production of sexed embryos, as well as in the prevention and control of genetic diseases [80].

Although the underlying mechanism is still not fully clear, research has already shown that the positive effects of melatonin include its antioxidant effects and its receptor-mediated signaling transduction. As an efficient antioxidant, melatonin is able to scavenge excess ROS from sperm, which may trigger DNA damage and sperm apoptosis. Melatonin also removes nitrogen-based reactants and toxic oxygen, as well as increases the activities of antioxidant enzymes, such as catalase (CAT), glutathione peroxidase (GPx), and superoxide dismutase (SOD). These effects are receptor-independent. Moreover, melatonin regulates Ca^2+^ signaling. Melatonin can activate the intracellular flow of Ca^2+^ into sperm, which helps to increase sperm motility. Melatonin also interacts with calmodulin, thereby influencing sperm cytoskeletal elements [66]. In addition, melatonin modulates second messenger cAMP levels, which can act both via the axoneme of the sperm tail and via cell membrane-dependent pathways to improve sperm mobility and velocity [66]. Melatonin receptors MT1 and MT2 receptors are expressed in sperm [72], and most of these effects are melatonin receptor-dependent (Table 1).

### 3.3. Melatonin Receptors, Reproductive Rhythm, and Endocrine Function

The biological rhythms of mammals are mainly controlled by the suprachiasmatic nucleus (SCN) of the hypothalamus. It receives light signals from the retina and regulates the circadian rhythms of organs such as the gonads through neuromodulation and humoral regulation. The circadian clock system plays an important role in the biological activities of the gonads, including the ovary and testis, and it is involved in the regulation of steroid hormone synthesis, oocyte maturation, ovulation, and seasonal estrus [60,81]. A disturbance in the biological clock can have an impact on gonadal function. SCN can regulate the secretion of melatonin from the pineal gland, and the local cells of the gonads can also secrete melatonin [81]. Melatonin receptors are widely distributed in the gonads, including the granulosa cells of the ovary and Leydig cells of the testis, which can participate in the regulation of the gonadal clock.

Melatonin acts on the HPG axis by regulating the hypothalamic gonadotropin, which can also bind directly to ovarian granulosa cells and have an effect on HPG [70]. Melatonin inhibits the expressions of gonadotropin-releasing hormone (GnRH) and GnRH receptors by upregulating the luteinizing hormone (LH) receptor. In turn, GnRH controls the secretion of the gonadotropins LH and follicle-stimulating hormone (FSH), which regulate reproductive function at the gonadal level and are involved in maintaining corpus luteum levels during pregnancy [70]. The wide distribution of melatonin receptors is the basis for its extensive biological effects, outside of the fact that melatonin acts as an antioxidant to prevent oxidative stress damage, which is receptor-independent [71]. In addition, rhythmic genes (Clock, Bmal1, Per2, and Cry1) are widely present in the HPG axis, in addition to the melatonin-related genes. Melatonin can regulate gonadal function by regulating the expression of rhythmic genes in different developmental stages of follicles [72] (Table 1).

Many animals exhibit distinct physiological and behavioral changes seasonally, and these adaptations have evolved to promote survival and reproductive success. Melatonin regulates seasonal aggression by altering both peripheral and neural steroidogenesis; aggressive behavior is well-conserved across animal taxa and enables individuals to compete with conspecifics for access to limited resources in their environment, such as food, territories, and mates [73]. Melatonin is crucial in affecting the relative reproductive mass in seasonally breeding species. There is evidence that the expression of the MT1 melatonin receptor affects the seasonally changing social behavior of male Siberian hamsters. MT1 receptor signaling in neural and peripheral tissues regulates the reproductive regulation of many seasonally reproducing species through the HPG reproductive axis [74]. However, there are some debates that the MT1 receptor can modulate seasonal changes in behavior, but not energetics or reproduction [56]. More research is still needed to reveal the mechanism linking melatonin receptors and animal reproduction (Table 1).

### 3.4. Melatonin Receptors and Embryonic Development

Melatonin affects cell proliferation and energy metabolism, and it plays a key role in embryo transfer and embryonic development. There is strong evidence that melatonin has a positive effect on in vitro embryo production (IVEP) and improves blastocyst quality in mammals, including bovine and sheep [82,83]. Mechanistic research has indicated that melatonin exerts its positive effect on oocyte competence, related not only to its antioxidant ability by scavenging ROS, but also to its increased mitochondrial activity and ATP levels [82]. The positive effect of melatonin-induced embryo quality improvement could be partly prevented by melatonin receptor antagonist luzindole [69]. During maternal pregnancy, melatonin can suppress the expression of proapoptotic genes, including Bax and Caspase-3, and activate the expression of the antiapoptotic gene Bcl-2, thus reducing the apoptosis rates of blastocysts and improving embryo quality and subsequent embryo implantation rate [84]. Knockdown experiments have shown that MT1 and MT2 demonstrate an antiapoptotic effect [7]. In contrast, another antioxidant (cysteamine) can also decrease ROS in oocytes and blastocysts, whereas it cannot improve blastocyst quality like melatonin. Altogether, it is demonstrated that melatonin receptors are partly responsible for the melatonin-induced positive effects on embryonic development.

## 4. Melatonin Receptors as Drug Targets in Therapy

It is challenging to assess the pharmacology and function of melatonin receptors, because their native binding site density in animal tissues is low or undetectable. Pharmacological research on melatonin receptors started in the late 1980s, when melatonin binding sites were detected using autoradiography with radiolabeled ligand 2-[^125^I]-iodomelatonin [14], which remains the main compound in identifying melatonin receptor-selective ligands [5,23] (Table 2). Moreover, tritiated melatonin ([^3^H]-melatonin) is another radioligand that is structurally closer to melatonin but with lower specific binding activity than 2-[^125^I]-iodomelatonin. Notably, these two radioligands work in different ways. Specifically, [^3^H]-melatonin has two melatonin-binding sites corresponding to the active state and inactive state in both MT1 and MT2 receptors, whereas 2-[^125^I]-iodomelatonin is only bound by activated melatonin receptors [85]. Novel iodinated radioligands such as SD6, an analogue of 2-[^125^I]-iodomelatonin, and DIV880 and S70254, MT2-selective radioligands, are promising ligands, although more work is still needed to confirm the applicability of these molecules [86] (Table 2). Furthermore, fluorescently labeled ligands are also promising compounds for investigating the pharmacology of melatonin receptors. The ligands described so far include the 7-azamelatonin analogue [87], coumarin-based compounds [88], and BODIPY-fused analogues [89,90]. Some of these compounds bind the MT1 and MT2 receptors non-selectively with good affinity (nM range), but data are scarce on their full pharmacological and functional properties (Table 2).

Competitive melatonin receptor antagonists are widely used in revealing the function of melatonin receptors. Luzindole, a non-selective antagonist for the MT1 and MT2 receptors, and 4-phenyl-2-propionamidotetralin (4P-PDOT), a selective MT2 antagonist with 300- to 1500-fold higher affinity for the MT2 receptor (Table 2), are considered the gold standards for characterizing membrane receptor-mediated effects and discriminating between MT1- and MT2-mediated effects [23]. In addition, *N*-butanoyl-2-(2-methoxy-6*H*-isoindolo [2,1-a]indol-11-yl)-ethanamine (IIK7) is a selective MT2 melatonin receptor agonist with higher affinity for the MT2 receptor in mouse or human [19] (Table 2). There remains a lack of reliable and efficient MT1-selective ligands. This is despite reports that the dimer S26131 formed by linking two agomelatine molecules has a more than 200-fold higher affinity for MT1 [91]; more data are still needed to confirm its pharmacology as a selective MT1 antagonist. Some studies also found an intriguing class of compounds, carbamate-derived insecticides (carbaryl and carbofuran), which are able to competitively bind melatonin receptors with μM affinity and influence receptor-mediated signaling [92] (Table 2). The pineal gland is affected by these compounds, resulting in changes in nocturnal plasma melatonin levels.

## 5. Conclusions and Perspectives

Melatonin is an important hormone in animals that regulates various physiological activities such as neuroendocrine function, regulation of seasonal reproduction, sexual maturation, immunoregulation, thermoregulation, some aspects of aging, and antioxidation. The various functions of the melatonin receptors are mainly transmitted through the activation of various signaling pathways. Many studies focused on melatonin-modulated animal reproduction and attempted to understand the melatonin receptor-mediated mechanisms. Although evidence has revealed that melatonin participates in the regulation of reproductive physiology, including rhythm regulation, seasonal behaver, gonadogenesis, gamete development, and maturation, there remains a lack of more in-depth and detailed research about the roles of melatonin receptors in their modulated signaling and their association with pathophysiology, as well as the screening of more efficient and specific receptor antagonists, especially MT1-selective antagonists. This can provide a bright future for the development of drugs and therapeutic methods based on melatonin receptors or receptor-mediated signaling pathways.

## Figures and Tables

**Table 1:** Study on melatonin receptors in animal reproductive regulation.

Tissue/Cells	Receptor Types	Functions	References
Leydig cells	Mainly MT1	Mediation of melatonin-inhibited steroid and androgen production	[58,59]
		Mediation of melatonin-stimulated CRH production and tyrosine phosphatase activity	[60]
Sertoli cells	MT1 and MT2	Mediation of the responsiveness to FSH during testicular development and regulation of spermatogenesis	[60]
		Mediation of melatonin-stimulated cell growth/proliferation and SSC proliferation	[63]
		Mediation of energy metabolism, such as lactate generation and glucose consumption	[64]
Sperm	MT1	Mediation of melatonin-inhibited apoptosis and improved fertilization ability	[65,66]
follicles	MT1 and MT2	Response to estrogen levels such as estradiol	[67,68]
Oocytes	MT1 and MT2	Mediation of melatonin-induced demethylation, including increase in Tet1 gene expression levels and decrease in Dnmt1 gene expression	[69]
Biorhythm	MT1 and MT2	Mediation of melatonin-regulated hormone secretion, including inhibition of GnRH, LH, and FSH	[70,71]
		Mediation of melatonin-regulated rhythmic gene expression, including Clock, Bmal1, Per2, and Cry1	[72]
		Related to seasonal variation in social behavior,	[56,73]
		modulating seasonal reproduction by activating or suppressing the HPG axis	[74]

MT1: melatonin receptor 1. MT2: melatonin receptor 2. GnRH: gonadotropin-releasing hormone. CRH: corticotropin-releasing hormone. FSH: follicle-stimulating hormone. LH: luteinizing hormone. SSCs: spermatogonial stem cells. HPG, hypothalamic–pituitary–gonadal.

**Table 2:** Pharmacological research on melatonin receptors.

Types	Name	Targeted Receptors	References
Radioligands	2-[^125^I]-iodomelatonin	Activated MT1 and MT2	[14]
	[^3^H]-melatonin	Active and inactive MT1 and MT2	[85]
Novel radioligands	SD6	MT1 and MT2	[86]
	DIV880	MT2	
	S70254	MT2	
Fluorescently labeled ligands	7-Azamelatonin	MT1 and MT2	[87]
	Coumarin-based compounds	MT1 and MT2	[88]
	BODIPY-fused analogues	MT1 and MT2	[89,90]
Competitive antagonists	Luzindole	MT1 and MT2	[23]
	4P-PDOT	MT2	
Agonist	IIK7	MT2	[19]
	Dimer S26131	MT1	[91]
Potential analogue	Carbamate-derived insecticides	MT1 and MT2	[92]

## Data Availability

Not applicable.
